# Supplementary material for: Salivary Protein Profile in Patients with Recurrent Aphthous Stomatitis: A Pilot Proteomic Study
Source: Int J Mol Sci. 2025 Aug 15;26(16):7878. doi: 10.3390/ijms26167878 (PMC12386313; doi:10.3390/ijms26167878)
Supplement: Supplementary file 1 [file ijms-26-07878-s001.zip › ijms-3817188-supplementary.pdf]

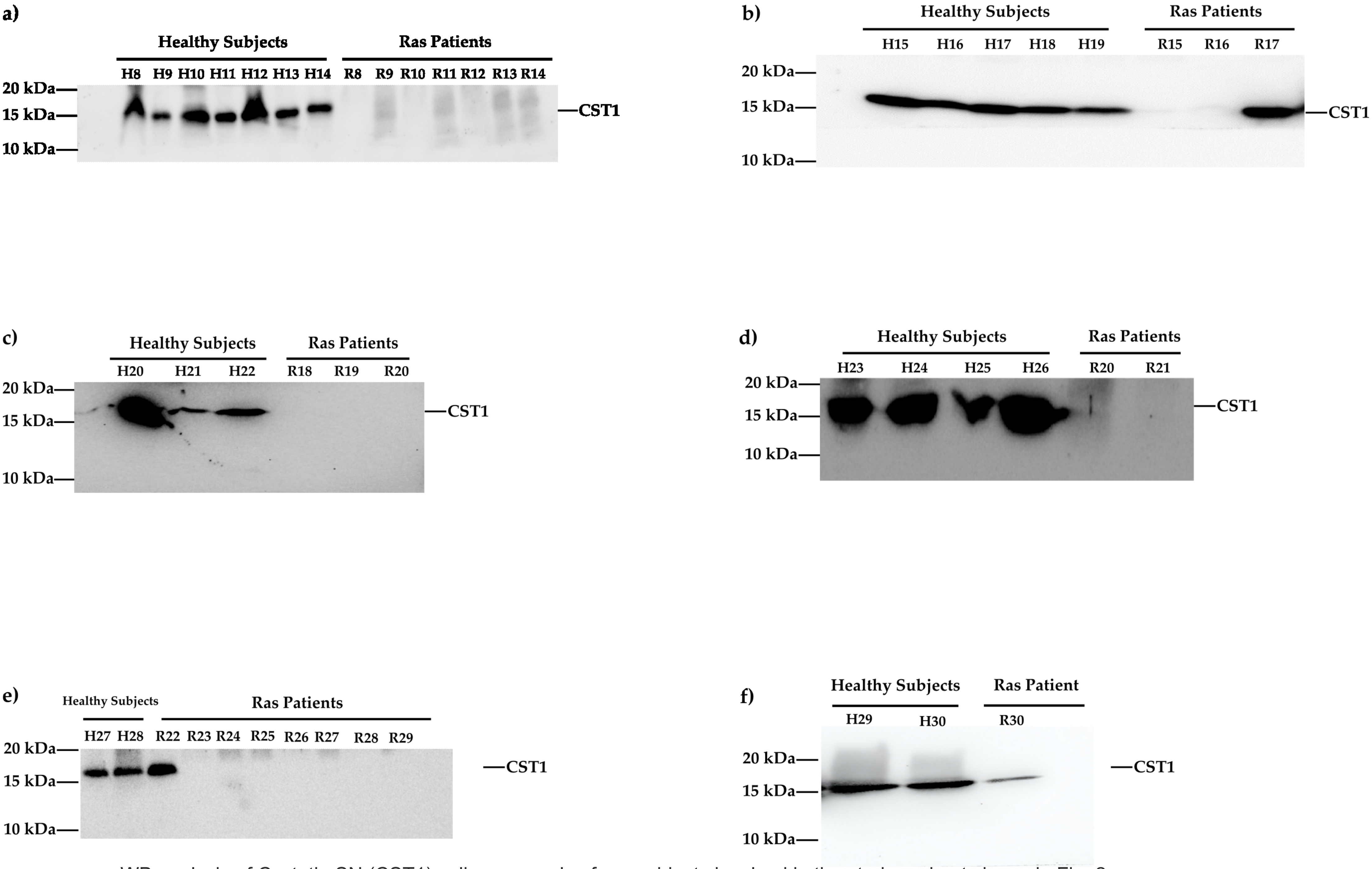

WB analysis of Cystatin SN (CST1) salivary samples from subjects involved in the study and not shown in Fig. 2. Healthy individuals (H) and Ras patients (R) were numbered from 1 to 30 for each group. WB of the samples from subjects 1–7 are shown in Fig. 2, while the remaining ones are divided into panels (a) to (f).
